# Role of released mitochondrial DNA in acute lung injury

**DOI:** 10.3389/fimmu.2022.973089

**Published:** 2022-08-18

**Authors:** Gangyu Long, Rui Gong, Qian Wang, Dingyu Zhang, Chaolin Huang

**Affiliations:** ^1^ Wuhan Jinyintan Hospital, Tongji Medical College of Huazhong University of Science and Technology, Wuhan, China; ^2^ The First Affiliated Hospital of University of Science and Technology of China (USTC), Division of Life Sciences and Medicine, University of Science and Technology of China, Hefei, China; ^3^ Hubei Clinical Research Center for Infectious Diseases, Wuhan, China; ^4^ Wuhan Research Center for Communicable Disease Diagnosis and Treatment, Chinese Academy of Medical Sciences, Wuhan, China; ^5^ Joint Laboratory of Infectious Diseases and Health, Wuhan Institute of Virology and Wuhan Jinyintan Hospital, Chinese Academy of Sciences, Wuhan, China

**Keywords:** ALI, ARDS, mtDNA, STING, TLR9, inflammasomes activation

## Abstract

Acute lung injury(ALI)/acute respiratory distress syndrome(ARDS) is a form of acute-onset hypoxemic respiratory failure characterised by an acute, diffuse, inflammatory lung injury, and increased alveolar-capillary permeability, which is caused by a variety of pulmonary or nonpulmonary insults. Recently, aberrant mitochondria and mitochondrial DNA(mtDNA) level are associated with the development of ALI/ARDS, and plasma mtDNA level shows the potential to be a promising biomarker for clinical diagnosis and evaluation of lung injury severity. In mechanism, the mtDNA and its oxidised form, which are released from impaired mitochondria, play a crucial role in the inflammatory response and histopathological changes in the lung. In this review, we discuss mitochondrial outer membrane permeabilisation (MOMP), mitochondrial permeability transition pore(mPTP), extracellular vesicles (EVs), extracellular traps (ETs), and passive release as the principal mechanisms for the release of mitochondrial DNA into the cytoplasm and extracellular compartments respectively. Further, we explain how the released mtDNA and its oxidised form can induce inflammatory cytokine production and aggravate lung injury through the Toll-like receptor 9(TLR9) signalling, cytosolic cGAS-stimulator of interferon genes (STING) signalling (cGAS-STING) pathway, and inflammasomes activation. Additionally, we propose targeting mtDNA-mediated inflammatory pathways as a novel therapeutic approach for treating ALI/ARDS.

## 1. Introduction

The Lung is a complex branched organ that terminates in a highly vascularised alveolus designed to efficiently exchange inspired oxygen with carbon dioxide (1). The alveolus, which takes up 90 percent of the total lung volume, consists of an epithelial layer of simple squamous epithelium, and an extracellular matrix surrounded by capillaries and is vulnerable to various extrapulmonary/indirect (e.g. sepsis, pancreatitis, trauma) or pulmonary/direct (e.g. pneumonia, aspiration) insults (2, 3). There has been growing evidence of diffuse alveolar damage in alveolar specimens taken from patients suffering from ARDS, and laboratory studies have documented epithelial and endothelial damage, which leads to the accumulation of protein-rich inflammatory fluids in the alveolar space (4). Excessive inflammatory fluids in the interstitium and alveoli cause impaired gas exchange, decreased compliance, and increased pulmonary arterial pressure. The annual rate of ARDS is generally 13–23 people per 100,000 in the general population (5), and is higher in intensive care units; approximately 10 to 15 percent of admitted patients and up to 23 percent of mechanically ventilated patients meet the criteria for ARDS (6), which may be underestimated seriously due to the ongoing COVID-19 pandemic. Over the past decades, great progress has been made in understanding the epidemiology, pathogenesis, and pathophysiology of ARDS, which is identified as a heterogeneous syndrome. Additionally, randomized trials have identified improved outcomes for patients with ARDS following the optimization of mechanical ventilation, fluid therapy, prone position ventilation, and neuromuscular blockade. However, none of these supportive therapies can reduce inflammation in the lungs nor have efficiently decreased the mortality of ALI/ARDS. According to the severity of lung injury (mild, moderate, and severe), the mortality rate remains high, approximately 27% to 45% (6), and has caused worldwide concern, especially in the COVID-19 pandemic, to establish more effective treatment strategies based on the pathogenesis to reduce the ALI mortality is urgently needed.

**Figure 1:**
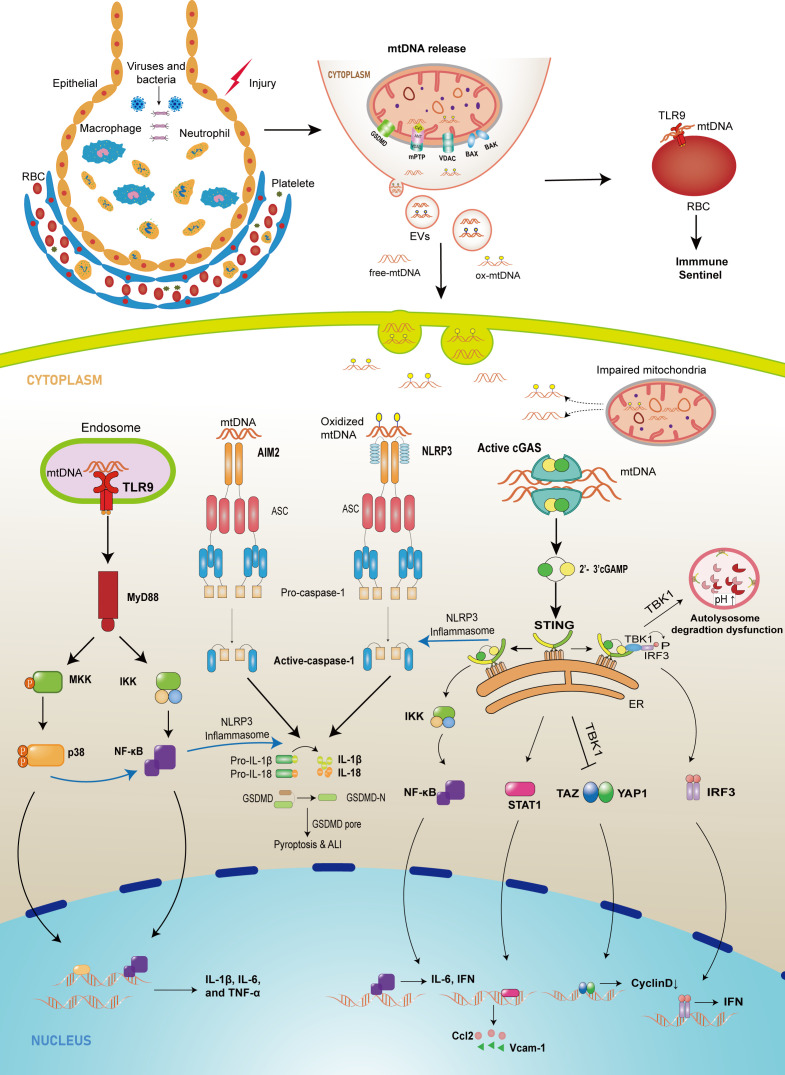
The schematic diagram of mtDNA-releasing and sensing pathways mediating injury in lung tissue. In cell injury or infection conditions, abnormal mtDNA can be released from mitochondria into cytosol through mPTP, GSDMD pore, MOMP mediated by BAX/BAK and VDAC, or from cytosol to extracellular environment by EVs. Once in the cytosol, mtDNA, which comes from intracellular injured mitochondria or extracellular space, can be recognized by three major sensors that drive the innate immune response. First, the released mtDNA can bind to TLR9 on the surface of erythrocytes to induce an immune response or bind to TLR9 in the endosome, which promotes the expression of downstream NF-κB or p38 MAPK, leading to an upregulation of pro-inflammatory factor expression. Cytoplasmic mtDNA is also recognized by cGAS and leads to increased expression of cytokines, adhesion molecules, and chemokines through different pathways and can inhibit autophagy and endothelial cell proliferation. In addition, the released mtDNA activates PRRs such as NLRP3 and AIM2, recruiting ASC and procaspase-1 to form inflammasomes and promoting IL-1β and IL-18 maturation and pyroptosis.

Mitochondria are the powerhouses of cells, which generate a large portion of adenosine triphosphate (ATP) through oxidative phosphorylation depending on the electron transport chain (ETC). In addition to their canonical function, mitochondria also play a role in immune modulation against infectious and sterile insults (7). Furthermore, mitochondria are closely associated with cellular integrity, alveolar surfactant secretion, and repair of alveolar epithelial and endothelial barriers (8–10). Mitochondrial dysfunction is a crucial causative factor that leads to pathological features of lung injury, such as inflammatory cell infiltration, explosive release of inflammatory cytokines, microvascular hyperpermeability, release of mitochondrial damage-associated molecular patterns (mtDAMPs), increased reactive oxygen species (ROS) production, and mitochondrial DNA damage (8, 11–16). However, several studies demonstrate that mitochondrial DNA (mtDNA), a mtDAMP, is significantly associated with ALI development under various stimuli (17–21). Furthermore, mtDNA obtained from bronchoalveolar lavage fluid (BALF) in animal models, and patients is associated with ALI development (22–25). Although evidences suggest some accessible ways to effectively protect against ALI by eliminating mitochondrial mtDNA (26–30), the mechanisms by which mitochondrial mtDNA promotes ALI and ARDS are complex and require further study. Thus, in this review, we summarize the possible mechanisms of mtDNA release, the pathogenic mechanisms of mtDNA in ALI/ARDS, as shown in **Figure 1**, and prospective therapeutic directions for ALI/ARDS.

## 2. Released mtDNA serves as a danger-associated molecular pattern in ALI/ARDS

Human mitochondrial DNA (mtDNA), localised in the mitochondrial matrix, comprises 16.5 kb circular dsDNA lacking introns and codes for 22 tRNAs, two rRNAs, and 13 mRNAs that encode the essential oxidative phosphorylation (OXPHOS) protein subunits (31). However, according to the mitochondrial endosymbiotic theory, which explains mitochondrial morphology (structurally similar to bacteria) and that mitochondria have their genetic code (32, 33), the unmethylated CpG and formylated peptides from mitochondrial DNA can be recognized as “foreign molecules” by our bodies after escaping from mitochondria (34–36). Thus, mtDNA is gradually recognized as a damage-associated molecular pattern (DAMP), a cellular component of dying cells, which contributes to initiating and propagating inflammation (37, 38). Moreover, under various stimuli such as bacterial or viral infections, trauma, and burns, mtDNA can be released into the cytoplasm and extracellular environment. As a newly identified mitochondrial DAMP in the last decade, mtDNA has attracted increasing attention in clinical and basic research, including ALI studies.

In the early stages of ALI/ARDS, mitochondrial dysfunction is observed in different types of ALI/ARDS, including infection, trauma, and burns (39, 40). Extracellular mtDNA in the blood and BALF of patients with ALI/ARDS has recently been investigated. As reported, mtDNA copies measured in the peripheral blood were associated with 28-day survival in patients with ARDS and poor outcomes in COVID-19 patients (41, 42). After multiple transfusions, mtDNA can also enter the receiver’s circulation and probably contributes to ALI/ARDS (43, 44).

Furthermore, circulating mtDNA in the blood has been suggested as a promising biomarker in the clinical context of ALI/ARDS (38, 45–48). Apart from circulating mtDNA, BALF mtDNA levels were significantly increased in patients with ALI/ARDS and were positively correlated with ALI progression (25). Moreover, numerous *in vivo* and *in vitro* experiments have shown the critical role of mtDNA in promoting ALI progression (49–52). Animal experiments have confirmed that mtDNA plays a critical role in ALI/ARDS models by activating the TLR9/MyD88/NF‐κB signalling pathway (18). Moreover, the administration of isolated mtDNA into the lungs or circulation induces the production of pro-inflammatory mediators (22, 29, 53). To further prove the hazardous potential of mtDNA, suppressing mtDNA levels by cyclosporine-A has been shown to alleviate ALI in LPS-induced ALI models (26).

The above evidence suggests that extracellular mtDNA is a significant damage factor during ALI progression and could serve as a promising predictor of ALI. Furthermore, strategies targeting mtDNA-related methods have been prospectively tested for treating ALI (26, 53).

The innate immune system is crucial in mediating injuries to alveolar epithelial cells and capillary endothelial cells in ALI/ARDS. During ALI/ARDS, elevated cytokines produced by innate immune pathways are pivotal factors in the deterioration of histopathological changes and lung function (54–56). Due to the unique features of mtDNA, it can trigger the activation of the innate immune system in numerous pathological conditions such as sepsis, ALI/ARDS, kidney injury, heart failure, and liver failure (18, 57–60). In terms of the wide distribution of mtDNA and its hazard potential, there is an urgent need to determine the comprehensive role of mtDNA in ALI/ARDS.

## 3. Mechanisms of mtDNA release in ALI/ARDS

Given the important role of mtDNA in the development of ALI/ARDS, we review the possible molecular mechanisms triggering the release of the mitochondrial genome into the cytoplasm and extracellular space as follows.

### 3.1 Mechanisms of mtDNA releasing into the cytosol

#### 3.1.1 mtDNA release mediated by MOMP

MOMP is a highly regulated process responsible for activating caspases and initiating apoptosis, usually controlled through the interactions between pro- and anti-apoptotic members of the B cell lymphoma 2 (BCL-2) family (61). This process is initiated by intrinsic apoptotic stimuli, such as DNA damage or endoplasmic reticulum (ER) stress, and mediated by BCL-2-associated X (BAX) and BCL-2 antagonist killer (BAK) to release intermembrane proteins into the cytosol (61). Then BAX/BAK oligomers induce the formation of macropores in mitochondria, which allows the herniation of the inner mitochondrial membrane (IMM) from these pores, resulting in the loss of membrane integrity and the subsequent release of the mitochondrial content, including mtDNA, into cytosol (62). Additionally, research has found that following MOMP, when the mitochondrial inner membrane is extruded through the permeabilized outer membrane, mitochondrial inner membrane permeabilization (MIMP) can then occur, allowing mtDNA egress into the cytosol (63). Recently, Li and colleagues found that severe fever with thrombocytopenia syndrome virus (SFTSV) infection triggers BAK upregulation and BAX activation in THP-1 cells, leading to mtDNA oxidization and subsequent cytosolic release (64). Additionally, ALI models induced by LPS in mice are characterized by an increased expression of BAK and BAX, indicating the participation of MOMP mediated by BAK/BAX in ALI/ARDS (65).

In addition, the mechanism of mtDNA release can also be mediated by voltage-dependent anion channels (VDACs) oligomers. VDACs are a family of pore-forming proteins discovered in the mitochondrial outer membrane and have the ability to form large mitochondrial outer membrane(MOM) pores, which are BAX/BAK-independent MOMP pathway. In mammals, three different isoforms have been described, namely VDAC-1, VDAC-2, and VDAC-3, which have different functions (66). Human VDAC-1 appears to be the most abundant isoform, and its oligomerization is associated with Cyto c release and apoptosis induction (67). In MEFs, Kim et al. reported that mtDNA fragment release requires VDAC oligomerization, and direct mtDNA–VDAC1 interactions are likely to increase VDAC oligomerization and mtDNA fragment release (68). Furthermore, in rats’ ALI models induced by paraquat, VDAC and caspase-3, -8, and -9 are significantly increased, together with acute diffuse damage of alveolar capillary endothelial cells, alveolar epithelial cells, and interstitial cells, indicating that VDAC plays a role in MOMP (69).

#### 3.1.2 mtDNA release mediated by mPTP

mPT is a phenomenon in which the accumulation of Ca2+ in the mitochondrial matrix makes IMM permeable and poorly selective (70). The mPTP is comprised of VDAC, adenine nucleotide translocase (ANT), and cyclophilin D (CyD) which is a permanent ingredient and modulator of mPTP (71). Moreover, alternating mitochondrial membrane potential, elevated Ca2+ level, oxidative stress, thiol oxidation, or altered pyridine nucleotide status could modulate mPTP opening (71). In 2004, Patrushev et al. firstly showed that the opening of the mPT pore could trigger the release of DNA fragments from mitochondria (72). Subsequently, a similar result was confirmed, the opening of the mPTP allowing the release of mtDNA fragments into the cytoplasm in multiple models, including immune cells, induced by irradiation, oxidative stress, lipopolysaccharide and extracellular ATP (70). Moreover, Fonai and colleagues discovered that the loss of this essential protein improves survival rate and intensely ameliorates LPS-induced lung injury in CyD(-/-) mice through inhibition of pro-inflammatory cytokine release, redox-sensitive cellular pathways such as MAPKs, Akt, and NF-κB activation, and the production of ROS (73). These data demonstrate that CyD-dependent mPT plays a crucial role in mitochondrial DAMPs (including mtDNA) release to inhibit inflammation and disease progression in ALI/ARDS.

### 3.2. Mechanisms of mtDNA releasing into extracellular space

#### 3.2.1 Release of mtDNA outside the cells through EVs

EVs, a heterogeneous group of cell-derived membranous structures comprising exosomes, microvesicles (MVs) (74, 75), and apoptotic EVs (ApoEVs) (76), are considered a mode of intercellular communication, acting as transport cargo of different messengers, including nucleic acids (mitochondrial and genomic DNA, non-coding RNA) and proteins (74, 77)

Exosomes are a subset of EVs with a size range of 40 to 160 nm (average 100 nm around) in diameter with an endosomal origin (78).Lázaro-Ibáñez and colleagues identified the existence of mtDNA in exosomes from the human mast cell line HMC-1 and erythroleukemic cell line TF-1 with sequencing analysis (79). Microvesicles (MVs), whose dimensions generally range from 50 to 500 nm, are circular fragments of membranes released from the endosomal compartment or shed from the surface membranes of most cell types (80), which have been shown to contain mtDNA from some contexts such as Streptococcus pneumonia (81). Apoptotic bodies (ApoBDs) with dimensions ranging between 1~5µm, a major subset of ApoEVs generated by apoptotic cells during the final stages of apoptosis (76), are formed through a process termed apoptotic cell disassembly characterized by a series of tightly regulated morphological steps including plasma membrane blebbing, apoptotic membrane protrusion formation, and fragmentation into ApoBDs (76)[05]. Although a recent study has demonstrated the existence of mtDNA in apoptotic bodies from acute myeloid leukaemia cells (82), more investigations in ALI/ARDS are urgently demanded.

Till now, growing evidence shows EVs not only participate in many physiological processes but also contribute to the pathogenesis of some diseases (83). In ALI/ARDS, alveolar macrophages (AMs)-derived MVs may play a significant role in the regulation of ALI-associated inflammation since macrophages were the major source of MVs in bronchoalveolar lavage fluid (84). Additionally, a recent study demonstrated that lung epithelial EVs or AM-EVs are responsible for the development of lung inflammation after sterile (hyperoxia) or infectious(P. pneumonia and S. pneumonia) stimuli in mice, respectively (85). However, due to the complex composition of the Evs, it is still difficult to confirm whether all components(including mtDNA) of the EVs can exacerbate lung damage, which needs further investigation.

#### 3.2.2 Release of mtDNA outside the cells through ETs

Recently, growing evidence has associated Extracellular Traps (ETs) with mtDNA, indicating ETs may participate in the process of mtDNA exteriorization. Neutrophil extracellular traps (NETs) are studied with great enthusiasm. Following strong activation signals, neutrophils release granule proteins and chromatin that form extracellular fibres that bind Gram-positive and -negative bacteria, shaping the so-called NETs (86). Moreover, NETs formation can be found in trauma or operation patients and are made of mtDNA without nuclear DNA component (87). However, the origin of mtDNA is a mystery. In LPS-induced ALI mice models, NETs produced by the recruited neutrophils can promote lung injury and inflammation, which can be rescued by NET inhibitors (88). Moreover, in ALI/ARDS models caused by influenza pneumonitis, neutrophils from infected lungs strongly induced NETs generation and augmented endothelial damage (89). In patients with severe pneumonia or COVID-19 acute respiratory distress syndrome, the plasma NETs level is also increased and associated with ARDS severity and mortality (90, 91). Although some evidence confirms the mtDNA release from immunocytes to form ETs, the release mechanisms are still unclear. Moreover, due to the direct evidence of mtDNA in ALI/ARDS ETs being scarce, the role of mtDNA from ETs in ALI/ARDS models and patients demands further investigation.

#### 3.2.3 Passive release of mtDNA

Passive release of mtDNA is mainly induced by cell death. In current knowledge, the main forms of cell death associated with ALI/ARDS can be concluded into apoptosis, necrosis, and pyroptosis (92). The most well-characterized and prevalent form of controlled cell death is apoptosis. According to different initiators in apoptosis pathways, the process can be summarized as the extrinsic and intrinsic pathways, the latter known as the mitochondrial pathway of apoptosis. During the intrinsic pathway, stimuli such as toxins, hypoxia, viral infections, and free radicals cause changes in the IMM and open the mPTP, causing mitochondrial components, including mtDNA and cytochrome c, to be released into the cytoplasm (93). ApoBDs are formed during the final stages of apoptosis and then released into the extracellular space with mtDNA and other materials inside.

Unlike apoptosis, necrosis is an alternative uncontrolled cell death induced by external injury, such as hypoxia or inflammation. This process often involves upregulating various pro-inflammatory proteins and compounds, such as nuclear factor-κB. The rupture of the cell membrane causes the spillage of the cell contents(including mtDNA) into surrounding areas during necrosis, resulting in a cascade of inflammation and tissue damage (92). Numerous studies have shown that circulation-free mtDNA level is associated with the extent of necrosis or damage, indicating that mtDNA is released from the necrotic cells into plasma (94, 95). In addition, in gastric aspiration-induced mice ALI models, mtDNA from cell-free bronchoalveolar lavage fluid increased 120-fold, indicating acid-induced cellular necrosis release numerous mtDNA and may augment lung injury (95).

Except for apoptosis and necrosis, pyroptosis also participates in the process of mtDNA release. Pyroptosis is a pro-inflammatory form of regulated cell death that relies on the enzymatic activity of inflammatory proteases that belong to the cysteine-dependent aspartate-specific protease (caspase) family and the subsequent cleavage of gasdermins (GSDMs) by different caspases (96). The latest study has found that GSDMs mediate the cellular release of mtDNA during pyroptosis or intrinsic apoptosis (97). Carlos and colleagues confirmed that GSDMDNT (amino-terminal of gasdermins D) contributes to mitochondrial DNA relocalization to the cytosol and GSDMENT (amino-terminal of gasdermins E) facilitates the cellular release of mtDNA in bone marrow-derived macrophages, HEK293T cells, and mice. In the development of ALI/ARDS, pyroptosis may be more common than other types of cell death due to uncontrolled progressive lung inflammation, and pyroptosis in different types of cells such as macrophages, neutrophils, alveolar epithelial cells, and capillary endothelial cells may play vital roles in lung injury and excessive inflammation (97).

## 4. Pathogenic mechanisms of mtDNA in ALI/ARDS

During the development of ALI/ARDS, dysfunctional mitochondria in the injured endothelium, epithelium, and recruited leukocytes generate large amounts of reactive oxygen species (ROS) and release mtDNA. Excessive ROS can disturb protein folding and structure and cause mitochondrial DNA mutations (98). Damaged mitochondrial genomes may disrupt aerobic respiration, cause cellular dysfunction, and even cell death. Moreover, the released mtDNA can contribute to the activation of the innate immune response through mtDNA sensors. Accumulating evidences suggest that mtDNA can be recognized by different mtDNA sensors (34, 99). Various mtDNA sensors and damaged mtDNA have been reported to play vital roles in ALI progression, as discussed below.

### 4.1 The TLR9 signaling

Toll-like receptors (TLRs) are critical components of innate pattern recognition receptors. To date, 12 TLRs have been identified in mice, and ten have been found in humans. Among these receptors, TLR9 is currently the only receptor that recognizes mtDNA with hypomethylated CpG motifs (100). TLR9 belongs to the type I integral membrane glycoprotein family with an N-terminal ligand recognition domain, a single transmembrane helix, and a C-terminal cytoplasmic signalling domain. It is mainly present in the endoplasmic reticulum (ER) membrane of unstimulated cells. When stimulated by CpG DNA, TLR9 translocates to the membrane of endosomes, where it recognizes its ligands and initiates cellular activation (101). After binding to mtDNA, TLR9 signalling can proceed through the adaptor myeloid differentiation primary response protein 88 (MyD88) to activate nuclear factor-κB (NF-κB) and through mitogen-activated protein kinases (MAPKs) to trigger inflammatory responses (34, 102). MAPKs include extracellular signal-regulated kinases (ERKs), c-Jun amino-terminal kinases (JNKs), and p38. p38 MAPKs are vital regulators of inflammatory cytokine expression and are activated by various stimuli (103). The transcription factor NF-κB plays its most important and evolutionarily conserved role in the immune system by regulating the expression of inducers and effectors at multiple points in the immune response (104).

Previous studies have revealed that mtDNA activates p38 MAPK after CpG-TLR9 interaction to induce inflammation in neutrophils (105, 106). Injury caused by cell-free mtDNA *via* the TLR9-p38 MAPK pathway has also been confirmed in some organs (59, 107, 108). In ALI, *in vivo* experiments demonstrated that TLR9 contributes to the development of ALI and systemic inflammation, and inhibition of TLR9 prominently attenuated histopathological changes and pro-inflammatory mediators, including interleukin-1 beta (IL-1β), interleukin-6 (IL-6), and tumor necrosis factor–α (TNF-α) (50, 109–114). Meanwhile, enhanced levels of mtDNA have been found in BALF and plasma from ALI patients and animal models (22, 25, 115). Additional administrations of mitochondrial DNA into the trachea or peritoneum aggravate systemic inflammation and lung injury in a TLR9-dependent manner *via* P38 MAPK activation (109, 110). Moreover, inhibition of TLR9 or p38 MAPK attenuated this effect, proving that extracellular mtDNA could mediate lung injury through the TLR9-p38 MAPK pathway (52, 110, 116, 117).

In addition, studies have found that mtDNA can induce NF-κB activity through TLR9 to regulate the expression of pro-inflammatory cytokine genes. According to these studies, mtDNA can increase NF-κB, IκB-α, and TLR9 expression in macrophage (118), and CPG-TLR9 activates NF-κB by regulating MyD88 to increase the production of pro-inflammatory mediators, such as TNF, IL-6, and adhesion molecules in lung epithelial cells (101, 119, 120). Furthermore, MyD88 knockout markedly reduced the levels of inflammatory cytokines and NF-κB and attenuated ALI (121). Suppression of TLR9 also partly decreases the production of inflammatory mediators by the TLR9-NF-κB pathway in alveolar epithelial cells (102, 122, 123). In addition to the single mechanisms described above, CpG DNA can promote collaborative activation of p38 MAPK and NF-κB in type I IFN-independent induction of IRF-7, CXCL10, and CCL3 to enhance IFN-α gene expression in human plasmacytoid dendritic cells (124). Additionally, although exogenous DNA from bacteria and viruses can activate interferon regulatory factors in a TLR9-dependent manner to enhance type I IFN responses, the role of mtDNA in this pathway still lacks strong evidence.

Interestingly, as conventional oxygen deliverers, red blood cells have been found to have a novel function: immune sentinels. Hotz et al. in 2018 found that red blood cells harbor a type of molecular sensor, TLR9, that recognizes and sticks to DNA molecules containing CpG motifs (125). *In vivo* experiments revealed a critical role of red blood cells in regulating systemic inflammation by scavenging cell-free mtDNA and demonstrated the importance of RBC-mediated DNA scavenging in limiting lung injury (125). Mangalmurti et al. in 2021 then explored the mechanisms by which RBCs binding with mtDNA through TLR9 appeared beneficial during quiescent states, where it promotes the scavenging of trace CpG to prevent nonspecific inflammation. During conditions characterized by excess circulating CpG, such as sepsis and COVID-19, binding CpG by RBC-TLR9 may lead to inflammation, and anaemia (126). Altogether, mtDNA recognition by TLR9 on RBCs provides evidence that RBCs are immune sentinels (127). However, the exact mechanism of this pathway requires further investigation.

### 4.2 The cGAS-STING pathway

Cyclic GMP-AMP synthase (cGAS) is a cytoplasmic DNA sensor that detects self-and non-self-DNA to synthesize cGAMP in a DNA-dependent manner (128, 129). cGAMP, a cyclic dinucleotide, is a second messenger usually generated by cells following DNA transfection or DNA virus infection (128). These cGAMPs bind to and activate STING to induce type I IFNs production. Stimulator of interferon genes (STING, also known as MITA, MPYS, ERIS, and TMEM173), a transmembrane protein located in the endoplasmic reticulum (ER) membrane, functions as a direct sensor of cGAMP and an essential signalling adaptor linking the interferon response to cytosolic DNA (130). Initially, studies illustrated that cytosolic DNA induces type I IFNs through the endoplasmic reticulum membrane protein STING, which subsequently activates the transcription factors NF-kB and IRF3 through the kinases IKK and TBK1, respectively (131–133). However, the mechanism upstream of STING activation is not fully understood. Subsequently, the cGAS-cGAMP-STING pathway was found to be essential for DNA-mediated immune response irrespective of cell type or DNA sequence, filling the gaps missing upstream of STING (134–137).

Activation of the cGAS-STING pathway begins with DNA recognition. DNA binds cyclic GMP-AMP synthase (cGAS) in a nonsequence-dependent manner through its phosphate backbone to form a 2:2 cGAS: DNA complex (138). Subsequently, cGAS generates a unique cGAMP, characterized by two phosphodiester bonds: one between the 2’OH of GMP and the 5’phosphate of AMP and the other between the 3’ OH of AMP and the 5’ phosphate of GMP, hereafter referred to as cGAMP (139). This novel class of second messengers is recognized by the ligand-binding pocket of STING and binds with hydrophobic interactions and hydrogen bonds (138), inducing an extensive conformational change in STING that is postulated to release a carboxy-terminal tail (CTT) that recruits and activates TBK1 (137). After binding to cGAMP, STING exits the ER in the form of vesicles and is transferred to the Golgi, where two cysteine residues of STING (Cys88 and Cys91) undergo palmitoylation, essential for STING oligomerization (140). STING recruits TANK-binding kinase (TBK1) and activates transcription factors IRF3 and NF-κB, inducing the production of type I interferons and pro-inflammatory cytokines (137, 141–143). Additionally, substantial evidence indicates that the cGAS-STING pathway also leads to dysfunctions in autophagy and endothelial proliferation (144–146).

Previous studies on STING have mainly focused on the immune response mediated by foreign nuclear acids from DNA viruses, retroviruses, bacteria, and parasites. Owing to the lack of pathogen-specific attributes, cGAS can participate in the antimicrobial defense and recognize self-DNA, including genomic and mitochondrial DNA. Cellular DNA is usually confined to the nucleus or mitochondria. However, in response to specific cellular stress or environmental insults, mitochondrial DNA is more susceptible to oxidative damage than nuclear DNA (nDNA) because of the incomplete DNA repair capacity in mitochondria and the proximity of mtDNA to the respiratory chain, which is the source of ROS production (34). Recent evidence has shown that mtDNA release and the cGAS-STING pathway can drive lung injury, making this pathway a promising therapeutic target for ALI (147). Activation of the cGAS-STING pathway plays a significant role in ALI/ARDS models and patients (51, 148, 149). Specifically, CMA (10-carboxymethyl-9-acridanone), a species-specific agonist of STING in mice, can promote type I IFN production by recognizing STING (150). DMXAA (5,6-dimethylxanthenone-4-acetic acid), another STING agonist in mice, can bind to STING and activate the TBK1-IRF-3 signalling pathway inducing IFN-β production (151). Recently, Togbe et al. found that the synthetic, non-nucleotidyl STING agonist diABZI induced type I IFN and pro-inflammatory mediators in bone marrow-derived macrophages and human airway epithelial cells.

Furthermore, endotracheal administration of diABZI also increased IFNα and IFNβ levels in BALF and triggered neutrophilic inflammation, accompanied by a substantial increase in mtDNA (149). Then, they treated mice receiving local airway diABZI with DNase I, efficiently abrogating dsDNA in the BALF and reducing neutrophil recruitment and neutrophil extracellular trap (NET) formation in the airways, while the levels of type I IFN and pro-inflammatory mediators were barely affected (149). In addition, antagonists of STING, such as C-178 and C-176 for mouse STING and H-151 for human STING, have been shown to inhibit the palmitoylation of STING, reduce TBK1 phosphorylation, and abrogate type I IFNs and IL-6 (152, 153). In addition, recent studies have revealed that recruited macrophages in the lung suppress alveolar macrophage-STING signalling *via* sphingosine kinase-2 (SPHK2)-mediated generation of sphingosine-1-phosphate (S1P), thereby mitigating inflammatory signalling and vascular injury (154, 155). Furthermore, as upstream stimulators of the cGAS-STING pathway, mtDNA and oxidized mtDNA were confirmed to play an essential role in enhancing immune and inflammatory responses (156–158). Li Liu et al. revealed that endogenous mtDNA and oxidized mtDNA could induce the formation of NETs depending on the TLR9 and STING pathways in bone marrow neutrophils from C57BL/6 mice then, the effect was abrogated by TLR9 knockout, STING knockout and reactive oxygen species scavenger (156). Additionally, Qing et al. reported that mtDNA in the cytoplasm triggered pyroptosis in a cGAS-STING-dependent manner, and the NLRP3 inflammasome participated in downstream effects to induce the production of IL-1β and IL-18 in mice (158). However, they did not reveal a connection between TBK1-IRF3 and NLRP3 activation in the downstream effects of the cGAS-STING pathway.

Besides the above mechanism, autophagy dysfunction has reached a stage recently (159–161). Ren et al. confirmed that autophagy dysfunction triggered by mtDNA could promote sepsis-related ALI *via* STING. They revealed that mtDNA contributed to disordered lysosomal acidification *via* STING, which caused deficient autophagic flux, and aggravated the disease in mice macrophages. Moreover, they revealed that STING-mediated autophagic flux blockade is partially associated with TBK1 downstream signalling (51). In addition, downstream signalling of the cGAS-STING pathway can suppress vascular regeneration to promote inflammatory lung injury. Huang et al. discovered that cGAS/STING activated by the released mtDNA inhibited endothelial proliferation by suppressing YAP signalling in HEK293 cells, human and mouse lung microvascular endothelial cells. Moreover, mtDNA-induced activation of TBK1 activated LATS1, inducing YAP phosphorylation and degradation, thereby preventing downstream YAP-mediated endothelial proliferation and repair (162). Interestingly, STING influences the mechanical barrier repair of endothelial cells and their paracrine function by over-expressing adhesion molecules and chemokines, which can organise the recruitment of immune cells and regulate leukocyte extravasation at places of inflammation. Wu et al. discovered that STING inhibition alleviated LPS-induced ALI in mice by suppressing vascular endothelial cell-mediated immune cell chemotaxis and adhesion. They revealed that a STING inhibitor or STING knockdown downregulated the expression levels of adhesion molecules and chemokines by decreasing the phosphorylation of the transcription factor STAT1 in HMEC-1cells (153).

### 4.3 Activation of inflammasomes

Inflammasomes are large multimolecular complexes consisting of receptor and sensor molecules, an adaptor protein (apoptosis-associated speck-like protein containing a CARD [ASC]), and the inflammatory cysteine protease caspase-1. There are five central receptors contributing to the formation of an inflammasome, including NOD, LRR, pyrin domain-containing protein 1 (NLRP1), NLRP3, NLR family CARD domain-containing protein 4 (NLRC4), absent in melanoma 2 (AIM2), and pyrin (163). Most of these receptors are activated by exogenous pathogen-associated molecular patterns (PAMPs) that gain access to the cytosol during microbial infection and endogenous DAMPs released from damaged or dying cells. Activated inflammasomes then activate the proteolytic enzyme caspase-1, which conversely regulates the proteolytic maturation of IL-1β and IL-18, as well as a quick, noxious, and inflammatory form of cell death termed pyroptosis (164). Recent studies have revealed the role of mtDNA in inducing caspase-1-mediated innate immune responses through the NLRP3 and AIM2 inflammasomes.

NLRP3 inflammasome is one of the most studied inflammasomes and is generally located in the cytoplasm. The NLRP3 inflammasome can be activated by a wide range of exogenous and endogenous stimuli such as lipopolysaccharide, nigericin, asbestos, alum, and extracellular ATP. In 2011, mtDNA was first revealed to contribute to NLRP3 inflammasome activation, confirming that upon LPS and ATP stimulation, mitochondrial ROS and mitochondrial membrane permeability increase prominently, causing the release of mtDNA into the cytoplasm. The translocated mtDNA requires activation of NLRP3 and could directly induce downstream activation of caspase-1 in response to treatment with LPS and ATP to enhance the production of IL-1β and IL-18 (165). Then, Shimada et al. discovered that oxidized mtDNA, generated and released into the cytosol during apoptosis, can bind to NLRP3 and activate the NLRP3 inflammasome, which 8-OH-dG competitively inhibits. They also demonstrated that NLRP3 is preferentially activated by oxidized DNA and AIM2 by normal DNA (166). Subsequently, Zhong and colleagues also confirmed that different activators of NLRP3 cause a specific form of mitochondrial damage that results in fragmented mtDNA release and increased production of ROS that is responsible for the conversion of mtDNA to an oxidized form, to be used as the ultimate NLRP3 ligand (167). However, the process of NLPR3 inflammasome activation induced by cytosolic mtDNA and extracellular mtDNA may differ. Li Ning et al. discovered that the cystolic mtDNA released from self-mitochondria contributed to LPS-induced ALI by upregulating cGAS, activating STING, and activating NLRP3 in mice. They also revealed that cGAS or STING deficiency relieved NLRP3-mediated pyroptosis of macrophages, and NLRP3 overexpression abolished the protective roles of STING deficiency in macrophages, indicating that the activation of NLRP3 is essential for the proinflammatory responses of STING (158). Nevertheless, the mechanism by which extracellular mtDNA induces the activation of the NLRP3 inflammasome is slightly different. Wu et al. first revealed the specific mechanism by which extracellular mtDNA in the lung induces NLRP3 inflammasome priming and activation through TLR9- p38 MAPK- NF-κB in THP-1 macrophages. They showed that knockdown of TLR9 offsets mtDNA-induced NLRP3 upregulation, decreases extracellular mtDNA-induced NLRP3 inflammasome transcription, and suppresses the production of mtDNA-induced IL-1β and TNF-α. This is consistent with the inhibition of p38 MAPK or NF-κB (52). Together with these studies, this shows that after the body is subjected to various external stresses, mitochondria are damaged and mtDNA is released into the cytoplasm. When cystolic mtDNA further activates the NLRP3 inflammasome in the cell, pyroptosis may be induced, and more mtDNA can be released outside the cell, causing the activation of the NLRP3 inflammasome in the peripheral cells, which may aggravate diseases such as ALI/ARDS.

AIM2 functions as a cytoplasmic dsDNA sensor and has been studied for over a decade. It binds DNA, recruits the inflammasome adaptor ASC, and activates caspase-1 (168, 169). Studies have found that the C-terminal HIN-200 domain interacts with the dsDNA sugar-phosphate backbone in a non-sequence-specific DNA recognition pathway, whereas the PYD domain of AIM2 associates with the adapter molecule ASC to activate both NF-κB and caspase-1 (170–172). Furthermore, activated caspase-1 promotes the maturation of IL-1β and IL-18 and cleaves gasdermin D, which can induce pyroptosis by forming pores on cellular membranes. Recently, Li et al. revealed that DNA from NETs, which accumulates in the lysosomes of alveolar macrophages under LPS treatment, binds with the AIM2 sensor to trigger the AIM2 inflammasome inducing the production of proinflammatory cytokines and macrophage pyroptosis (173). The study also found that the degradation of NET DNA or silencing of the AIM2 gene can alleviate pyroptosis in alveolar macrophages co-stimulated with LPS and NETs. Although NETs have been identified as an important source of extracellular mtDNA (87, 174, 175), the interaction between mtDNA from NETs and AIM2 sensors in lung injury has not been specifically illustrated. Further studies are needed to explore the interactions involved in ALI pathogenesis.

In addition, some studies have provided evidence of mtDNA binding to NLRC4 complexes. For example, in 2015, Jabir et al. discovered that mitochondrial DNA could bind specifically to NLRC4 immunoprecipitates and that the transfected mtDNA could directly activate the NLRC4 inflammasome in BMDMs from mice and HEK cells (176). They also revealed that inflammasome activation was enhanced by DNA oxidation and downregulated by autophagy. However, it is still challenging to demonstrate the role of the mtDNA-NLRC4 interaction in lung injury owing to the lack of direct evidence.

## 5. mtDNA in ALI/ARDS caused by bacterial and viral pneumonia

Bacterial and viral pneumonia account for the major cases of ARDS in recent clinical trials. Increasing evidence showed bacterial and viral infection could induce mitochondria damage and mtDNA release (64, 81, 162, 177–181). Once leaving its original location, the mtDNA can be recognized as foreign, eliciting a potent innate immune response. Nerlich et al. found that the pneumolysin produced by Streptococcus pneumoniae (S.pn.) could cause mtDNA to release through mPTP opening and transfer into extracellular space *via* microvesicles, which may contribute to S.pn. related inflammatory lung injury (81). Meanwhile, Huang et al. demonstrated that LPS(Lipopolysaccharide), the main pathogenic component of Gram-negative bacteria, activated the pore-forming protein Gasdermin D, which formed mitochondrial pores and induced mitochondrial DNA (mtDNA) release into the cytosol of endothelial cells, suppressing endothelial cell proliferation by cGAS-YAP signalling pathway and promoting inflammatory injury (162). Additionally, upon different viral infections, including cytomegalovirus (MCMV) and Sendai virus (SeV), both of which are associated with the ALI/ARDS development (182, 183), vaccinia virus-related kinase two is associated with voltage-dependent anion channel 1 (VDAC1) and promoted VDAC1 oligomerization and mtDNA release, leading to the cGAS-mediated innate immune response (178). Moreover, Moriyama and colleagues discovered that the cytosolic mtDNA release induced by Influenza A virus can be partially inhibited by the knockdown of Bax and induce cGAS/STING-dependent IFN-β gene expression

(177), indicating the critical role of Bax/Bak for mtDNA release into the cytosol after the influenza virus infection which can lead to ARDS, a fatal complication (184). Although studies have now elucidated the release of mitochondrial DNA and the immune response generated by some bacteria and viruses, further exploration of common pathogens causing ALI/ARDS is still urgently needed due to the large heterogeneity between different pathogens.

## 6. Prospects for treatment in ALI/ARDS

As the release of mtDNA and its oxidized form are the common priming step for mtDNA-sensing pathways mediating excessive immune responses, protective strategies specific to mtDNA or mitochondria may be preferred choices for the treatment of ALI/ARDS. Xian et al. demonstrated Metformin inhibits mtDNA synthesis, abrogating NLRP3 inflammasome activation and pulmonary inflammation in LPS and SARS-CoV-2 induced mice ARDS models (185). Additionally, prevention of mtDNA release can also prominently alleviate lung injury. Reversely, exogenous administration of mtDNA can provoke lung injury and systemic inflammation (109).

Based on the essential role of mtDNA at the onset and progression of ALI/ARDS, accessible therapeutic targets can be divided into three main aspects: inhibiting the release of mtDNA, clearing the released mtDNA, and impeding mtDNA-mediated pathways. We conclude with the following possible therapeutic methods.

In terms of mtDNA release mechanisms, the reduction of mtDNA release primarily focuses on scavenging ROS and maintaining mitochondrial homeostasis. For example, Chao et al. found that peroxyauraptenol, isolated from the seeds of Cnidium monnieri, could inhibit inflammation and NLRP3 inflammasome activation by reducing mitochondrial ROS levels, indicating a possible candidate for anti-inflammatory agents (186). Recently, Sok et al. found that 1′-cetoxychavicol acetate, a natural compound from the tropical ginger Alpinia species, is a promising inhibitor of the NLRP3 inflammasome *via* mitochondrial ROS suppression (187). Additionally, dexmedetomidine and tyrosine phosphatase SHP2 have been shown to preserve mitochondrial dynamic equilibrium and homeostasis respectively, to attenuate inflammation (188, 189). In addition, some possible agents can limit mtDNA release with unknown mechanisms, such as cyclosporine A, epigallocatechin gallate, α7 nicotinic acetylcholine receptor signalling, and metformin (26, 53, 185, 190, 191). Some of these agents can inhibit inflammasome activation to alleviate inflammation by limiting the release of mtDNA.

Autophagy is the most studied target in the process of eliminating the released mtDNA. Several studies support the crucial role of autophagy in eliminating and degrading mitochondrial DNA (59, 165, 192, 193). Moreover, many studies have revealed potent agents that can reinforce autophagy to mitigate inflammatory injuries, such as Apelin-13, carbon monoxide, genipin, and transcription factor EB (194–197). Furthermore, some studies have found that enhanced mitophagy can have similar effects in alleviating lung injury. These agents include polydatin, redox, and Sestrin 2 (198–200). In addition, DNaseI targeting mtDNA shows treatment potential in paraquat-induced ALI and pulmonary fibrosis, which needs further investigation in other kinds of ALI (201).

Regarding mtDNA-mediated pathways, regulating the levels of released mtDNA can indirectly abrogate downstream pathways. The possible agents are principally studied in the innate immune pathogenic pathways described above to explore the direct inhibiting mechanisms, which may not always be completely explained by the downstream pathways and require further investigation. Such agents, including carbon monoxide, epigallocatechin-3-gallate, riboflavin, Tanreqing, and ethyl pyruvate, need to be explored further (28, 202–205).

## 7. Conclusion

For ALI/ARDS development, growing evidence associates mitochondrial dysfunction with impaired metabolism and pathological changes, leading to different levels of alveolar inflammation and pulmonary edema (11, 12, 23, 206). Among the mitochondrial DAMPs from impaired mitochondria, mtDNA has shown a high correlation with the severity of ALI/ARDS and has great potential to promote lung inflammatory responses in clinical and basic research. Therefore, We summarize the main mechanisms for releasing mitochondrial DNA into the cytoplasm and extracellular compartments, such as MOMP, mPTP, Evs, ETs and passive release. Moreover, we also discuss the released mtDNA and its oxidized form can bind to DNA sensors, such as TLR9, cGAS, NLRP3, AIM2, and NLRC4, to induce lung injury and the production of proinflammatory cytokines. According to the vital role of mtDNA in ALI/ARDS, eliminating released mtDNA, inhibiting the release of mtDNA, or disturbing mtDNA-mediated pathways have shown prominent therapeutic effects, indicating that therapies targeting released mtDNA are promising. However, the mechanisms underlying mtDNA-mediated pathways in ALI/ARDS must be explored. Moreover, strategies targeting mtDNA-associated lung injury must be optimized and made more efficient.

## Author contributions

CH and DZ generated the research idea and finally revised the manuscript. GL, RG and QW wrote the general manuscript. All authors contributed to the article and approved the submitted version.

## Funding

This work was supported by a grant from the National Natural Science Foundation of China (92169107).

## Conflict of interest

The authors declare that the research was conducted in the absence of any commercial or financial relationships that could be construed as a potential conflict of interest.

## Publisher’s note

All claims expressed in this article are solely those of the authors and do not necessarily represent those of their affiliated organizations, or those of the publisher, the editors and the reviewers. Any product that may be evaluated in this article, or claim that may be made by its manufacturer, is not guaranteed or endorsed by the publisher.
